# Synthesis and crystal structure of [(*S_p_*)-(2-phenyl­ferrocen­yl)meth­yl]tri­methyl­ammonium iodide di­chloro­methane monosolvate

**DOI:** 10.1107/S2056989022006053

**Published:** 2022-06-14

**Authors:** Abdelhak Lachguar, Eric Deydier, Agnès Labande, Eric Manoury, Rinaldo Poli, Jean-Claude Daran

**Affiliations:** a CNRS, LCC (Laboratoire de Chimie de Coordination), Université de Toulouse, UPS, INPT, 205 Route de Narbonne, F-31077 Toulouse Cedex 4, France; b IUT A Paul Sabatier, de Chimie, Avenue Georges Pompidou, CS 20258, F-81104, Castres Cedex, France

**Keywords:** 1,2-disubstitued ferrocene, planar chirality, chiral ligands, asymetric catalysis, disordered di­chloro­methane, crystal structure

## Abstract

As a follow-up to our research on the chemistry of disubstituted ferrocene derivatives, the synthesis and the structure of the title compound, [Fe(C_5_H_5_)(C_15_H_19_N)]I·CH_2_Cl_2_, is described. The cation mol­ecule is built up from a ferrocene disubstituted by a tri­methyl­ammonium methyl group and a phenyl ring. The asymmetric unit contains the iodide to equilibrate the charge and a disordered di­chloro­methane solvate. The disordered model results from a roughly statistical exchange (0.6/0.4) between one Cl and one H. The packing of the structure is stabilized by weak C—H⋯*X* (*X* = I, Cl), C—H⋯π(Cp) and C—Cl⋯π(phen­yl) inter­actions, building a three-dimensional network. The cation has planar chirality with *S_p_* (Fc) absolute configuration. The structure of the title compound is compared with related disubstituted (tri­meth­ylammonio)­methyl ferrocenes.

## Abstract 2

The packing of the title disubstituted ferrocene derivative is stabilized by weak C—H⋯*X* (*X* = I, Cl), C—H⋯π(Cp) and C—Cl⋯π(phen­yl) inter­actions, building a three-dimensional network. The cation has planar chirality with *S*_p_(Fc) absolute configuration. The structure of the title compound is compared with related disubstituted (tri­meth­ylammonio)­methyl ferrocenes.

## 1. Chemical context

Asymmetric catalysis by transition metals has received considerable attention over the last few decades and numerous chiral ligands and complexes allowing high activity and enanti­oselectivity have been reported (Jacobsen *et al.*, 1999 ▸; Börner, 2008 ▸). For this purpose, catalysts need a chiral ligand presenting at least a chiral center, a chiral axis or a planar chirality. Amongst the various chiral ligands that have been synthesized, ferrocenyl phosphines have proven to be particularly efficient for numerous asymmetric reactions (Buergler *et al.*, 2012 ▸; Gómez Arrayás *et al.*, 2006 ▸; Toma *et al.*, 2014 ▸)

Over the last few years, our team has developed the synthesis of various chiral ferrocenyl ligands for asymmetric catalysis (Audin *et al.*, 2010 ▸; Labande *et al.*, 2007 ▸; Bayda *et al.*, 2014 ▸; Daran *et al.*, 2010 ▸; Wei *et al.*, 2012 ▸, 2014 ▸; Loxq *et al.*, 2014 ▸). We mainly focused on a series of chiral bidentate P*X* ferrocenyl ligands (*X* = O*R*, S*R*, NHC) bearing planar chirality, which have been successfully used in different homogeneous asymmetric catalytic reactions: allylic substitution, meth­oxy­carbonyl­ation, hydrogenation (Kozinets *et al.*, 2012 ▸; Le Roux *et al.*, 2007 ▸; Diab *et al.*, 2008 ▸; Routaboul *et al.*, 2005 ▸). All of these ligands present a planar chiral 1,2-disubstituted ferrocenyl group with coordination sites on both substituents. More recently, we wanted to extend the application of planar chiral 1,2-disubstituted ferrocenyl groups to the synthesis of ligands with only one substituent bearing a coordination site for fine tuning of existing ligands. To this aim, we needed an enanti­omerically pure planar chiral building block bearing a good leaving group in order to introduce a planar chiral substituent on nucleophilic atoms. In this context, we report here the two-step synthesis of the title [(*S_p_*)-(2-phenyl­ferrocen­yl)meth­yl]tri­methyl­ammonium iodide salt. 
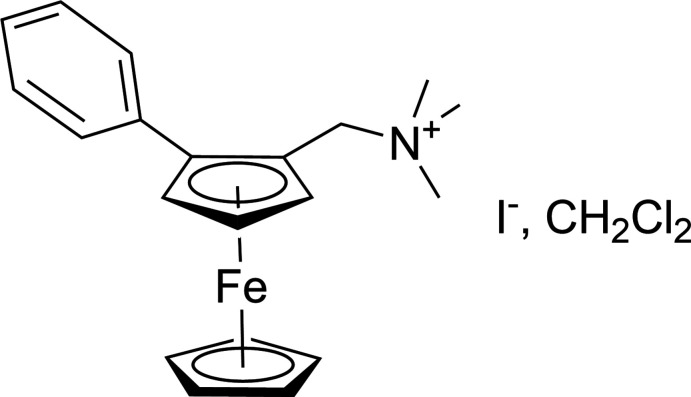


**Figure 1:**
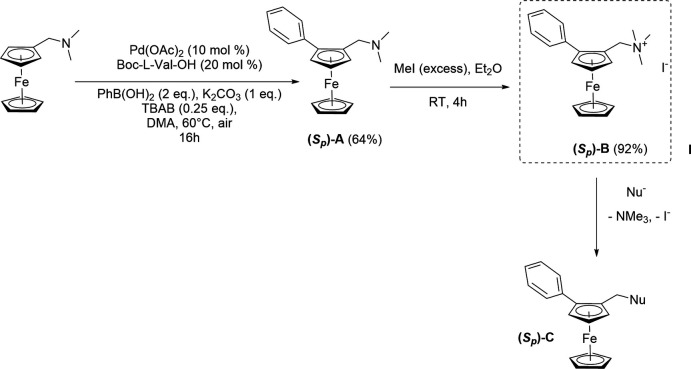
Synthesis of the title (*S_p_*)-1-di­methyl­amino­methyl-2-phenyl­ferrocenium iodide salt

The latter is synthesized in two steps, the first consists in the enanti­oselective synthesis of (*S_p_*)-A following the procedure developed by S.-L. You and co-workers (Gao *et al.*, 2013 ▸), the second step is a quaternization of the tertiary amine to the ammonium salt by reaction with an excess of iodo methane. (Ferro­cenyl­meth­yl) ammoniums have been used successfully as electrophiles because of the stabilization of carbocations in an α position of ferrocene derivatives and because of the presence of a good leaving group: tri­methyl­amine. Nucleophilic[EM2] substitution (Lin *et al.*, 2020 ▸) on the methyl­ene carbon atom in the α position of the ferrocene moiety in compound B, [(*Sp*)-(2-phenyl­ferrocen­yl)meth­yl]tri­methyl­ammonium iodide, should then be favoured and should provide an efficient access to a wide range of various enanti­omerically pure ferrocene deriv­atives of type C including ligands, by reaction with various nucleophiles (amines, thiols, alcohols) (Fig. 1 ▸).

## 2. Structural commentary

**Figure 2:**
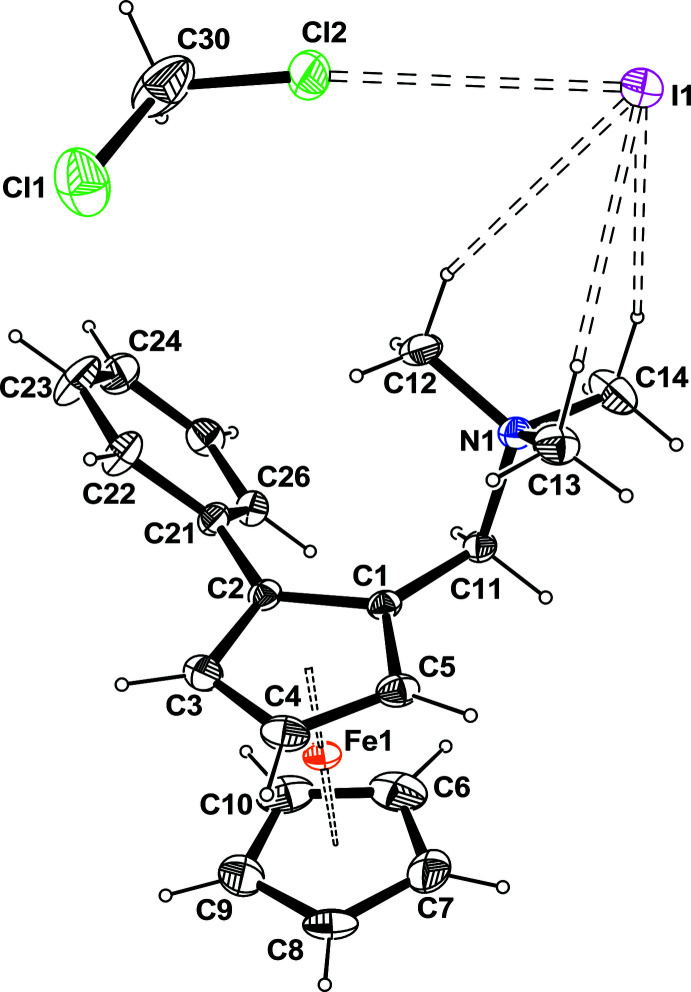
View of the asymmetric unit of the title compound with the atom-labelling scheme. Ellipsoids are drawn at the 30% probability level and the H atoms are represented as small circles of arbitrary radii. C—H⋯*X* (*X* = I, Cl) inter­actions are represented as dashed lines.

**Figure 3:**
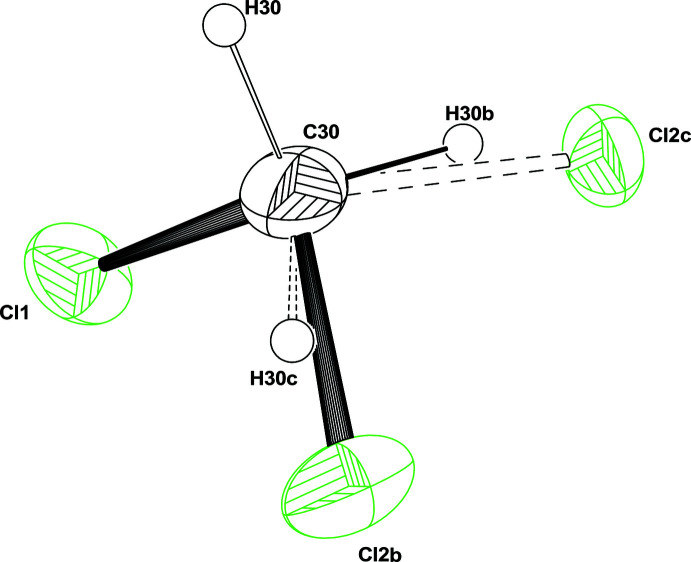
*ORTEP* view of the disordered CH_2_Cl_2_ solvent mol­ecule. Ellipsoids are drawn at the 20% probability level. H atoms are represented as small circles of arbitrary radii.

The mol­ecular structure is based on a ferrocene moiety in which one of the Cp rings is disubstituted in the 1,2 position by a tri-methyl­ammonium-methyl and a phenyl substituent. The mol­ecule has a positive charge, which is counterbalanced by an iodide (Fig. 2 ▸). Moreover, there is one disordered di­chloro­methane solvate mol­ecule per asymmetric unit. The disordered model results from the exchange between one Cl and one H in the ratio 0.6/0.4 (Fig. 3 ▸). This disorder might be induced by the occurence of weak C—Cl⋯I intra­molecular and C—H⋯Cl inter­molecular inter­actions. There are weak intra­molecular C—H⋯I inter­actions within the asymmetric unit.

As a result of the presence of the two substituents on the Cp ring, the cation mol­ecule has planar chirality and its absolute structure is *S_p_*, which is confirmed by the refinement of the Flack parameter (Parsons *et al.*, 2013 ▸). The phenyl ring is twisted with respect to the Cp ring by 48.74 (17)° and the C1–C11–N1 unit is roughly perpendicular to the Cp ring to which it is attached, making a dihedral angle of 89.7 (2)°.

## 3. Supra­molecular features

**Figure 4:**
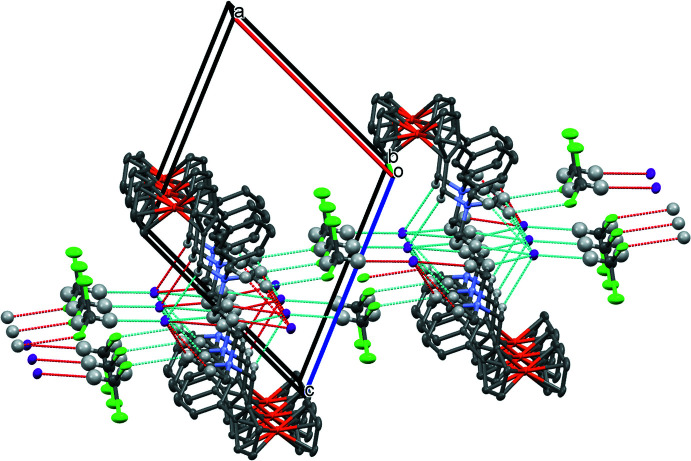
Partial packing view showing the C—H⋯*X* (*X* = I, Cl) inter­molecular inter­actions resulting in the formation of ribbons parallel to the *b* axis and C—H⋯Cl inter­actions linking the ribbons to form a layer parallel to ( 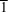 01) plane. The di­chloro­methane solvate builds the link between the layers.

The crystal packing is governed by the occurrence of weak C—H⋯*X* (*X* = Cl, I), C—H⋯π and C—Cl⋯π inter­actions (Table 1 ▸). The iodine atom is engaged in many weak C—H⋯I inter­actions involving some of the H atoms of the methyl groups, one H atom of the methyl­ene group and the non-disordered H atoms of the di­chloro­methane solvate. These inter­actions build up a ribbon developing parallel to the *b* axis (Fig. 4 ▸). Then the Cl2 atom of the chloro­form solvate inter­acts with the C12—H12*C* methyl group, thus building a link between the strips, resulting in a layer parallel to the ( 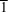 01) plane (Fig. 4 ▸). Moreover, there are two weak C—H⋯π inter­actions involving atom H13*B* of the C13 methyl group with the centroid of the Cp ring (C6–C10; *Ct*2) and atom C23 of the phenyl group with the centroid of the substituted Cp ring (C1–C5; *Ct*1). Finally, there is also a C—Cl⋯π inter­action involving the Cl1 atom of the solvate [C30—Cl1⋯*Ct*3 (C21–C26), 1.757 (8), 3.4096 (2) and 4.7694 (3) Å, 132.13 (1)°]. All these inter­actions build up a three-dimensional network.

## 4. Database survey

A search in the Cambridge Structural Database (version 5.36; Groom *et al.*, 2016 ▸) using a fragment containing a ferrocenyl disubsituted by a tri­methyl­amoniummethyl and at least a C atom gave six hits that could be compared with the title compound. A comparison of C1—C11, C11—N1 distances and dihedral angles between the Cp ring and the C1–C11–N1 plane is shown in Table 2 ▸. In all these compounds, the bulky N(CH_3_)_3_ group is always above the Cp ring to which it is attached. The dihedral angles between the Cp and the C–C–N plane range from 69.8 to 89.7° for the title compound.

## 5. Synthesis and crystallization

**Synthesis of (** ***S_p_*** **)-1-di­meth­ylamino­methyl-2-phen­ylferrocene [(** ***S_p_*** **)-A]:** To a solution of phen­ylboronic acid (110 mg, 1 mmol) in DMA (8 mL) were added Boc–*L*–Val–OH (43.5 mg, 0.2 mmol), Pd(OAc)_2_ (22.5 mg, 0.1 mmol), K_2_CO_3_ (138.21 mg, 1 mmol), TBAB (tetra­butyl ammonium bromide; 80 mg, 0.25 mmol) and *N*,*N*-di­meth­ylferrocen­ylmeth­ylamine (243 mg, 1 mmol) successively. The mixture was stirred at 333 K under air (open flask). When the reaction was complete (TLC monitoring), the mixture was quenched with saturated aqueous NaHCO_3_ and the organic phase was extracted three times with EtOAc. The combined organic layers were washed with H_2_O and brine successively, dried (Na_2_SO_4_) and filtered. The solvent was removed under reduced pressure and the residue purified by column chromatography (ethyl acetate/petroleum ether = 1/10, *v*/*v*, 2% Et_3_N) to afford the desired product A as a yellow oil (205 mg, 64% yield). The results are in agreement with published analytical data (Gao *et al.*, 2013 ▸).

^1^H NMR (400 MHz, CDCl_3_) δ ppm 7.79–7.71 (*m*, 2H, CH Ph), 7.41–7.31 (*m*, 2H, CH Ph), 7.30–7.21 (*m*, 1H, CH Ph), 4.53–4.47 (*m*, 1H, CH subst Cp), 4.33 (*dd*, *J* = 2.5, 1.5 Hz, 1H, CH subst Cp), 4.26 (*t*, *J* = 2.5 Hz, 1H, CH subst Cp), 4.08 (*s*, 5H, CH C_p_), 3.67 (*d*, *J* = 12.8 Hz, 1H, CH_2_), 3.18 (*d*, *J* = 12.8 Hz, 1H, CH_2_), 2.21 (*s*, 6H, CH_3_). ^13^C NMR (101 MHz, CDCl_3_) δ ppm 138.91 (C_q_, Ph), 129.36 (CH Ph), 127.93 (CH Ph), 126.06 (CH Ph), 88.17 (C_q_ subst Cp), 82.24 (C_q_ subst Cp), 71.56 (CH subst Cp), 70.06 (CH Cp), 69.94 (CH subst Cp), 67.10 (CH subst Cp), 57.93 (CH_2_), 45.08 (CH_3_).

**Synthesis of [(** ***Sp*** **)-(2-phenyl­ferrocen­yl)meth­yl]tri­methyl­ammonium iodide salt [(** ***S_p_*** **)-B]:** An excess of MeI (1 mL, 1.62 mmol) was added to a solution of **A** (250 mg, 0.78 mmol) in Et_2_O (3 mL). The reaction mixture was stirred for 4 h at RT. An abundant yellow solid precipitated. The yellow solid was filtered, washed with Et_2_O and dried to yield B as a yellow solid (332 mg, 92% yield), which was crystallized in di­chloro­methane.

^1^H NMR (400 MHz, CDCl_3_) δ ppm 7.58–7.50 (*m*, 2H, CH Ph), 7.46–7.37 (*m*, 2H, CH Ph), 7.37–7.26 (*m*, 1H, CH Ph), 5.33 (*d*, *J* = 13.4 Hz, 1H, CH_2_), 4.91 (*dd*, *J* = 2.5, 1.5 Hz, 1H, CH subst Cp), 4.83 (*d*, *J* = 13.4 Hz, 1H, CH_2_), 4.56 (*dd*, *J* = 2.5, 1.5 Hz,1H, CH subst Cp), 4.52 (*t*, *J* = 2.5 Hz, 1H, CH subst Cp), 4.34 (*s*, 5H, CH C_p_), 3.02 (*s*, 9H, CH_3_). ^13^C NMR (101 MHz, CDCl_3_) δ ppm 136.54 (C_q_, Ph), 129.86 (CH Ph), 129.05 (CH Ph), 127.72 (CH Ph), 90.61 (C_q_ subst Cp), 73.44 (CH subst Cp), 72.15 (C_q_ subst Cp), 70.96 (CH Cp), 70.44 (C_q_ subst Cp), 70.04 (C_q_ subst Cp), 65.21 (CH_2_), 52.76 (CH_3_).

## 6. Refinement

Crystal data, data collection and structure refinement details are summarized in Table 3 ▸. All H atoms attached to C atoms were fixed geometrically and treated as riding with C—H = 1.0 Å (methine), 0.95 Å (aromatic), 0.99 Å (methyl­ene) and 0.98 Å (meth­yl) with *U*_iso_(H) = 1.2U_eq_(CH aromatic, methyl­ene) or *U*_iso_(H) = 1.5*U*_eq_(CH_3_).

The occurrence of three large residual densities around the C atom of the solvate with distances around 1.76 Å initially suggested the presence of a chloroform solvate mol­ecule. However, if one of the Cl atoms (Cl1) could be refined correctly with full occupancy, the two others display large and elongated ellipsoids. Refining their occupancy factors using the restraints available in *SHELXL* gave a ratio of 0.6/0.4. So the disordered model is based on an exchange between one H and one Cl (Fig. 3 ▸). The model has been refined using the PART instruction to model two CH_2_Cl_2_ models. The non-disordered atoms C30, H30 and Cl1 were split with occupancy factor 0.5 and introduced in the two models (C30*A*, C30*B*, H30*A*, H30*B*, Cl1*A*, Cl1*B*). Their coordinates and thermal parameters were constrained to be identical using the EXYZ and EADP commands available in *SHELXL*. This disordered model is not perfect, as suggested by a large residual electron density in the vicinity of the atom H30*B*.

## Supplementary Material

Crystal structure: contains datablock(s) I, global. DOI: 10.1107/S2056989022006053/dj2043sup1.cif

Structure factors: contains datablock(s) I. DOI: 10.1107/S2056989022006053/dj2043Isup2.hkl

CCDC reference: 2177602

Additional supporting information:  crystallographic information; 3D view; checkCIF report

## Figures and Tables

**Table 1:** Hydrogen-bond geometry (Å, °)

*D*—H⋯*A*	*D*—H	H⋯*A*	*D*⋯*A*	*D*—H⋯*A*
C11—H11*A*⋯I1^i^	0.99	3.17	3.988 (4)	140
C12—H12*C*⋯I1	0.98	3.21	4.117 (5)	154
C12—H12*B*⋯Cl2*C*	0.98	3.51	3.787 (7)	99
C13—H13*A*⋯I1	0.98	3.27	4.158 (5)	151
C14—H14*A*⋯I1	0.98	3.14	4.057 (5)	157
C14—H14*B*⋯I1^i^	0.98	3.25	4.077 (5)	143
C30*A*—H30*A*⋯I1^ii^	1.00	2.89	3.867 (7)	166
C13—H13*B*⋯CT2^iii^	0.98	2.98	3.901 (6)	158
C23—H23⋯CT1^iv^	0.95	2.69	3.600 (7)	160

Symmetry codes: (i) 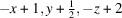 ; (ii) 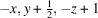 ; (iii) 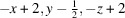 ; (iv) 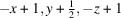 .

**Table 2:** Comparison of the geometry (Å, °) within the methyl­amine C–CH_2_–N fragment for the title compound with related structures.

	C1—C11	C11—N1	Cp1/C1>N1
Title compound	1.493 (5)	1.531 (4)	89.7 (2)
BECKUQ	1.509	1.534	85.6
LIFWUS	1.465	1.544	69.8
LIFWUS	1.509	1.536	78.6
LIFXAZ	1.494	1.525	70.4
PIJLEB	1.494	1.519	86.9
VIKZIA	1.485	1.538	86.0
XEQKIN	1.497	1.531	84.2

References: BECKUQ (Hitchcock *et al.*, 2002 ▸); LIFWUS (Malezieux *et al.*, 1994 ▸); LIFXAZ (Malezieux *et al.*, 1994 ▸); PIJLEB (Butler *et al.*, 2002 ▸); VIKZIA (Butler *et al.*, 2002 ▸); XEKQIN (Deck *et al.*, 2000 ▸).

**Table 3:** Experimental details

Crystal data	
Chemical formula	[Fe(C_5_H_5_)(C_15_H_19_N)]I·CH_2_Cl_2_
*M*_r_	546.08
Crystal system, space group	Monoclinic, *P*2_1_
Temperature (K)	110
*a*, *b*, *c* (Å)	10.7919 (7), 10.2128 (5), 11.2941 (7)
β (°)	113.031 (3)
*V* (Å^3^)	1145.57 (12)
*Z*	2
Radiation type	Mo *K*α
μ (mm^−1^)	2.24
Crystal size (mm)	0.30 × 0.10 × 0.10

Data collection	
Diffractometer	Bruker APEXII CCD
Absorption correction	Multi-scan (*SADABS*; Krause *et al.*, 2015 ▸)
*T*_min_, *T*_max_	0.520, 0.746
No. of measured, independent and observed [*I* > 2σ(*I*)] reflections	58547, 6993, 6822
*R*_int_	0.052
(sin θ/λ)_max_ (Å^−1^)	0.715

Refinement	
*R*[*F* ^2^ > 2σ(*F* ^2^)], *wR*(*F* ^2^), *S*	0.033, 0.092, 1.06
No. of reflections	6993
No. of parameters	247
No. of restraints	1
H-atom treatment	H-atom parameters constrained
Δρ_max_, Δρ_min_ (e Å^−3^)	1.50, −0.69
Absolute structure	Flack *x* determined using 3144 quotients [(*I* ^+^)−(*I* ^−^)]/[(*I* ^+^)+(*I* ^−^)] (Parsons *et al.*, 2013 ▸)
Absolute structure parameter	0.018 (6)

Computer programs: *APEX2* and *SAINT* (Bruker, 2015 ▸), *SHELXT* (Sheldrick, 2015*a* ▸), *SHELXL2018* (Sheldrick, 2015*b* ▸), *ORTEPIII* (Burnett & Johnson, 1996 ▸), *ORTEP-3 for Windows* (Farrugia, 2012 ▸) and *Mercury* (Macrae *et al.*, 2020 ▸).

## supplementary crystallographic information

### Crystal data

**Table:**

[Fe(C_5_H_5_)(C_15_H_19_N)]I·CH_2_Cl_2_	*F*(000) = 544
*M**_r_* = 546.08	*D*_x_ = 1.583 Mg m^−^^3^
Monoclinic, *P*2_1_	Mo *K*α radiation, λ = 0.71073 Å
*a* = 10.7919 (7) Å	Cell parameters from 9989 reflections
*b* = 10.2128 (5) Å	θ = 2.8–28.3°
*c* = 11.2941 (7) Å	µ = 2.24 mm^−^^1^
β = 113.031 (3)°	*T* = 110 K
*V* = 1145.57 (12) Å^3^	Stick, yellow
*Z* = 2	0.30 × 0.10 × 0.10 mm

### Data collection

**Table:**

Bruker APEXII CCD diffractometer	6993 independent reflections
Radiation source: micro-focus sealed tube	6822 reflections with *I* > 2σ(*I*)
Graphite monochromator	*R*_int_ = 0.052
φ and ω scans	θ_max_ = 30.5°, θ_min_ = 2.1°
Absorption correction: multi-scan (SADABS; Krause *et al.*, 2015)	*h* = −15→15
*T*_min_ = 0.520, *T*_max_ = 0.746	*k* = −14→14
58547 measured reflections	*l* = −16→16

### Refinement

**Table:**

Refinement on *F*^2^	Secondary atom site location: difference Fourier map
Least-squares matrix: full	Hydrogen site location: inferred from neighbouring sites
*R*[*F*^2^ > 2σ(*F*^2^)] = 0.033	H-atom parameters constrained
*wR*(*F*^2^) = 0.092	*w* = 1/[σ^2^(*F*_o_^2^) + (0.0507*P*)^2^ + 0.9038*P*] where *P* = (*F*_o_^2^ + 2*F*_c_^2^)/3
*S* = 1.06	(Δ/σ)_max_ = 0.001
6993 reflections	Δρ_max_ = 1.50 e Å^−^^3^
247 parameters	Δρ_min_ = −0.68 e Å^−^^3^
1 restraint	Absolute structure: Flack *x* determined using 3144 quotients [(*I*^+^)-(*I*^-^)]/[(*I*^+^)+(*I*^-^)] (Parsons *et al.*, 2013)
Primary atom site location: structure-invariant direct methods	Absolute structure parameter: 0.018 (6)

### Special details

**Table:**

Geometry. All esds (except the esd in the dihedral angle between two l.s. planes) are estimated using the full covariance matrix. The cell esds are taken into account individually in the estimation of esds in distances, angles and torsion angles; correlations between esds in cell parameters are only used when they are defined by crystal symmetry. An approximate (isotropic) treatment of cell esds is used for estimating esds involving l.s. planes.

### Fractional atomic coordinates and isotropic or equivalent isotropic displacement parameters (Å^2^)

**Table:**

	*x*	*y*	*z*	*U*_iso_*/*U*_eq_	Occ. (<1)
Fe1	0.94060 (5)	0.53142 (6)	0.79508 (5)	0.02716 (11)	
I1	0.22433 (3)	0.13532 (3)	0.79999 (3)	0.04532 (10)	
N1	0.5997 (3)	0.3408 (3)	0.8610 (3)	0.0281 (6)	
C1	0.7697 (3)	0.4388 (3)	0.7817 (3)	0.0243 (6)	
C2	0.7375 (3)	0.5512 (4)	0.6967 (3)	0.0248 (6)	
C3	0.8016 (4)	0.5304 (5)	0.6083 (4)	0.0323 (7)	
H3	0.797426	0.587679	0.540618	0.039*	
C4	0.8728 (4)	0.4085 (5)	0.6395 (4)	0.0361 (8)	
H4	0.924513	0.371724	0.596435	0.043*	
C5	0.8532 (4)	0.3521 (4)	0.7450 (4)	0.0316 (7)	
H5	0.888964	0.270870	0.784766	0.038*	
C6	1.0323 (5)	0.6035 (7)	0.9762 (5)	0.0588 (18)	
H6	0.996901	0.602449	1.041048	0.071*	
C7	1.1125 (5)	0.5035 (6)	0.9540 (6)	0.0546 (14)	
H7	1.139685	0.424007	1.000841	0.065*	
C8	1.1439 (4)	0.5440 (6)	0.8501 (6)	0.0482 (11)	
H8	1.196503	0.496477	0.814202	0.058*	
C9	1.0835 (5)	0.6679 (6)	0.8081 (6)	0.0517 (13)	
H9	1.088663	0.717959	0.739283	0.062*	
C10	1.0143 (5)	0.7038 (6)	0.8864 (7)	0.0542 (14)	
H10	0.964387	0.782051	0.879465	0.065*	
C11	0.7314 (3)	0.4163 (4)	0.8937 (3)	0.0262 (6)	
H11A	0.723121	0.502191	0.930581	0.031*	
H11B	0.804794	0.367396	0.960681	0.031*	
C12	0.4841 (4)	0.4146 (5)	0.7671 (6)	0.0463 (11)	
H12A	0.496041	0.423269	0.685738	0.069*	
H12B	0.479920	0.501808	0.801502	0.069*	
H12C	0.400292	0.367326	0.752068	0.069*	
C13	0.6057 (5)	0.2094 (4)	0.8064 (5)	0.0389 (9)	
H13A	0.518810	0.165131	0.783295	0.058*	
H13B	0.676498	0.156995	0.870448	0.058*	
H13C	0.625814	0.219443	0.729424	0.058*	
C14	0.5803 (6)	0.3216 (6)	0.9844 (5)	0.0501 (13)	
H14A	0.494379	0.277179	0.966431	0.075*	
H14B	0.579953	0.406942	1.023851	0.075*	
H14C	0.654042	0.268085	1.043360	0.075*	
C21	0.6496 (4)	0.6618 (3)	0.6930 (3)	0.0279 (7)	
C22	0.5483 (5)	0.6960 (5)	0.5744 (4)	0.0425 (10)	
H22	0.539718	0.649794	0.498588	0.051*	
C23	0.4595 (6)	0.7987 (7)	0.5682 (6)	0.0594 (16)	
H23	0.390870	0.820909	0.487518	0.071*	
C24	0.4694 (5)	0.8671 (5)	0.6747 (6)	0.0469 (11)	
H24	0.408025	0.935960	0.668760	0.056*	
C25	0.5712 (5)	0.8348 (5)	0.7930 (5)	0.0372 (8)	
H25	0.579600	0.882142	0.868150	0.045*	
C26	0.6603 (4)	0.7334 (4)	0.8012 (4)	0.0308 (7)	
H26	0.729624	0.712896	0.882019	0.037*	
C30A	0.1255 (9)	0.5103 (11)	0.4280 (8)	0.086 (3)	0.5
H30C	0.160640	0.586709	0.485441	0.103*	0.5
H30A	0.029108	0.526078	0.374882	0.103*	0.5
Cl1A	0.2146 (3)	0.4929 (2)	0.3278 (2)	0.0790 (6)	0.5
Cl2B	0.1425 (3)	0.3690 (3)	0.5204 (3)	0.0674 (8)	0.6
C30B	0.1255 (9)	0.5103 (11)	0.4280 (8)	0.086 (3)	0.5
H30D	0.032076	0.537089	0.374507	0.103*	0.5
H30B	0.121215	0.423883	0.465898	0.103*	0.5
Cl1B	0.2146 (3)	0.4929 (2)	0.3278 (2)	0.0790 (6)	0.5
Cl2C	0.1919 (5)	0.6201 (4)	0.5491 (4)	0.0680 (11)	0.4

### Atomic displacement parameters (Å^2^)

**Table:**

	*U*^11^	*U*^22^	*U*^33^	*U*^12^	*U*^13^	*U*^23^
Fe1	0.0183 (2)	0.0300 (2)	0.0302 (2)	−0.00341 (18)	0.00622 (17)	−0.0044 (2)
I1	0.03471 (13)	0.05047 (17)	0.04750 (16)	−0.00545 (12)	0.01253 (11)	0.00816 (14)
N1	0.0238 (13)	0.0291 (14)	0.0308 (15)	−0.0013 (11)	0.0099 (11)	−0.0001 (11)
C1	0.0183 (13)	0.0244 (15)	0.0269 (14)	−0.0016 (11)	0.0052 (11)	−0.0012 (12)
C2	0.0205 (13)	0.0268 (15)	0.0230 (13)	−0.0016 (12)	0.0041 (10)	−0.0013 (11)
C3	0.0294 (16)	0.0410 (19)	0.0267 (15)	−0.0056 (16)	0.0113 (13)	−0.0044 (15)
C4	0.0301 (17)	0.041 (2)	0.040 (2)	−0.0051 (15)	0.0170 (16)	−0.0133 (17)
C5	0.0237 (15)	0.0258 (16)	0.043 (2)	−0.0018 (12)	0.0102 (14)	−0.0055 (14)
C6	0.035 (2)	0.095 (5)	0.040 (2)	−0.026 (3)	0.0078 (18)	−0.027 (3)
C7	0.030 (2)	0.060 (3)	0.051 (3)	−0.014 (2)	−0.0078 (19)	0.011 (2)
C8	0.0196 (16)	0.059 (3)	0.062 (3)	−0.0088 (19)	0.0115 (17)	−0.013 (3)
C9	0.036 (2)	0.053 (3)	0.058 (3)	−0.020 (2)	0.010 (2)	0.003 (2)
C10	0.030 (2)	0.048 (3)	0.072 (4)	−0.0106 (19)	0.007 (2)	−0.024 (3)
C11	0.0221 (14)	0.0273 (15)	0.0253 (14)	−0.0034 (12)	0.0050 (12)	−0.0001 (12)
C12	0.0215 (16)	0.045 (2)	0.065 (3)	0.0037 (16)	0.0092 (18)	0.016 (2)
C13	0.036 (2)	0.0298 (19)	0.050 (2)	−0.0059 (15)	0.0161 (18)	−0.0073 (17)
C14	0.054 (3)	0.062 (3)	0.042 (2)	−0.023 (2)	0.027 (2)	−0.010 (2)
C21	0.0245 (14)	0.0288 (18)	0.0265 (14)	0.0016 (12)	0.0058 (12)	0.0030 (12)
C22	0.041 (2)	0.045 (2)	0.0309 (19)	0.0142 (19)	0.0026 (17)	0.0023 (17)
C23	0.050 (3)	0.064 (3)	0.046 (3)	0.030 (3)	−0.001 (2)	0.007 (2)
C24	0.044 (2)	0.041 (2)	0.057 (3)	0.018 (2)	0.021 (2)	0.010 (2)
C25	0.041 (2)	0.0325 (17)	0.044 (2)	0.0020 (16)	0.0226 (18)	−0.0021 (15)
C26	0.0319 (17)	0.0299 (17)	0.0298 (16)	0.0016 (14)	0.0112 (14)	0.0025 (13)
C30A	0.071 (5)	0.121 (8)	0.060 (4)	0.051 (5)	0.021 (3)	0.005 (4)
Cl1A	0.1058 (16)	0.0728 (11)	0.0816 (12)	0.0165 (10)	0.0615 (12)	0.0079 (9)
Cl2B	0.0526 (12)	0.107 (2)	0.0579 (13)	0.0290 (14)	0.0379 (11)	0.0377 (14)
C30B	0.071 (5)	0.121 (8)	0.060 (4)	0.051 (5)	0.021 (3)	0.005 (4)
Cl1B	0.1058 (16)	0.0728 (11)	0.0816 (12)	0.0165 (10)	0.0615 (12)	0.0079 (9)
Cl2C	0.105 (3)	0.0471 (19)	0.0525 (17)	0.005 (2)	0.0320 (19)	−0.0071 (15)

### Geometric parameters (Å, º)

**Table:**

Fe1—C1	2.025 (3)	C9—H9	0.9500
Fe1—C6	2.030 (5)	C10—H10	0.9500
Fe1—C7	2.034 (5)	C11—H11A	0.9900
Fe1—C8	2.037 (4)	C11—H11B	0.9900
Fe1—C5	2.037 (4)	C12—H12A	0.9800
Fe1—C10	2.038 (5)	C12—H12B	0.9800
Fe1—C9	2.041 (5)	C12—H12C	0.9800
Fe1—C2	2.043 (3)	C13—H13A	0.9800
Fe1—C4	2.048 (4)	C13—H13B	0.9800
Fe1—C3	2.055 (4)	C13—H13C	0.9800
N1—C12	1.488 (6)	C14—H14A	0.9800
N1—C13	1.489 (5)	C14—H14B	0.9800
N1—C14	1.500 (6)	C14—H14C	0.9800
N1—C11	1.529 (5)	C21—C26	1.390 (5)
C1—C5	1.435 (5)	C21—C22	1.402 (5)
C1—C2	1.449 (5)	C22—C23	1.404 (7)
C1—C11	1.495 (5)	C22—H22	0.9500
C2—C3	1.435 (5)	C23—C24	1.359 (9)
C2—C21	1.465 (5)	C23—H23	0.9500
C3—C4	1.433 (7)	C24—C25	1.398 (8)
C3—H3	0.9500	C24—H24	0.9500
C4—C5	1.412 (6)	C25—C26	1.391 (6)
C4—H4	0.9500	C25—H25	0.9500
C5—H5	0.9500	C26—H26	0.9500
C6—C10	1.400 (10)	C30A—Cl2B	1.748 (11)
C6—C7	1.422 (10)	C30A—Cl1A	1.758 (8)
C6—H6	0.9500	C30A—H30C	0.9900
C7—C8	1.406 (9)	C30A—H30A	0.9900
C7—H7	0.9500	C30B—Cl2C	1.695 (11)
C8—C9	1.418 (9)	C30B—Cl1B	1.758 (8)
C8—H8	0.9500	C30B—H30D	0.9900
C9—C10	1.411 (9)	C30B—H30B	0.9900
			
C1—Fe1—C6	108.39 (19)	C7—C6—Fe1	69.7 (3)
C1—Fe1—C7	119.30 (19)	C10—C6—H6	125.7
C6—Fe1—C7	41.0 (3)	C7—C6—H6	125.7
C1—Fe1—C8	153.2 (2)	Fe1—C6—H6	126.0
C6—Fe1—C8	68.1 (2)	C8—C7—C6	107.3 (5)
C7—Fe1—C8	40.4 (2)	C8—C7—Fe1	69.9 (3)
C1—Fe1—C5	41.37 (15)	C6—C7—Fe1	69.3 (3)
C6—Fe1—C5	126.6 (2)	C8—C7—H7	126.4
C7—Fe1—C5	106.4 (2)	C6—C7—H7	126.4
C8—Fe1—C5	117.9 (2)	Fe1—C7—H7	126.0
C1—Fe1—C10	127.2 (2)	C7—C8—C9	108.2 (5)
C6—Fe1—C10	40.3 (3)	C7—C8—Fe1	69.7 (3)
C7—Fe1—C10	68.5 (3)	C9—C8—Fe1	69.8 (3)
C8—Fe1—C10	68.3 (2)	C7—C8—H8	125.9
C5—Fe1—C10	164.6 (2)	C9—C8—H8	125.9
C1—Fe1—C9	164.8 (2)	Fe1—C8—H8	126.2
C6—Fe1—C9	67.8 (3)	C10—C9—C8	108.0 (5)
C7—Fe1—C9	68.3 (2)	C10—C9—Fe1	69.7 (3)
C8—Fe1—C9	40.7 (2)	C8—C9—Fe1	69.5 (3)
C5—Fe1—C9	152.8 (2)	C10—C9—H9	126.0
C10—Fe1—C9	40.5 (3)	C8—C9—H9	126.0
C1—Fe1—C2	41.75 (14)	Fe1—C9—H9	126.4
C6—Fe1—C2	120.6 (2)	C6—C10—C9	107.8 (5)
C7—Fe1—C2	155.2 (2)	C6—C10—Fe1	69.6 (3)
C8—Fe1—C2	163.3 (2)	C9—C10—Fe1	69.9 (3)
C5—Fe1—C2	69.89 (15)	C6—C10—H10	126.1
C10—Fe1—C2	108.24 (19)	C9—C10—H10	126.1
C9—Fe1—C2	126.2 (2)	Fe1—C10—H10	126.1
C1—Fe1—C4	68.89 (16)	C1—C11—N1	114.3 (3)
C6—Fe1—C4	163.1 (3)	C1—C11—H11A	108.7
C7—Fe1—C4	124.6 (2)	N1—C11—H11A	108.7
C8—Fe1—C4	106.3 (2)	C1—C11—H11B	108.7
C5—Fe1—C4	40.44 (18)	N1—C11—H11B	108.7
C10—Fe1—C4	154.4 (3)	H11A—C11—H11B	107.6
C9—Fe1—C4	119.1 (2)	N1—C12—H12A	109.5
C2—Fe1—C4	69.31 (16)	N1—C12—H12B	109.5
C1—Fe1—C3	69.09 (15)	H12A—C12—H12B	109.5
C6—Fe1—C3	155.3 (2)	N1—C12—H12C	109.5
C7—Fe1—C3	162.3 (2)	H12A—C12—H12C	109.5
C8—Fe1—C3	125.5 (2)	H12B—C12—H12C	109.5
C5—Fe1—C3	68.75 (18)	N1—C13—H13A	109.5
C10—Fe1—C3	120.5 (2)	N1—C13—H13B	109.5
C9—Fe1—C3	107.8 (2)	H13A—C13—H13B	109.5
C2—Fe1—C3	40.99 (14)	N1—C13—H13C	109.5
C4—Fe1—C3	40.88 (19)	H13A—C13—H13C	109.5
C12—N1—C13	108.7 (4)	H13B—C13—H13C	109.5
C12—N1—C14	110.2 (4)	N1—C14—H14A	109.5
C13—N1—C14	108.1 (4)	N1—C14—H14B	109.5
C12—N1—C11	110.9 (3)	H14A—C14—H14B	109.5
C13—N1—C11	111.6 (3)	N1—C14—H14C	109.5
C14—N1—C11	107.2 (3)	H14A—C14—H14C	109.5
C5—C1—C2	108.2 (3)	H14B—C14—H14C	109.5
C5—C1—C11	124.2 (3)	C26—C21—C22	118.3 (4)
C2—C1—C11	127.5 (3)	C26—C21—C2	123.4 (3)
C5—C1—Fe1	69.8 (2)	C22—C21—C2	118.3 (4)
C2—C1—Fe1	69.78 (19)	C21—C22—C23	119.8 (4)
C11—C1—Fe1	123.7 (2)	C21—C22—H22	120.1
C3—C2—C1	106.7 (3)	C23—C22—H22	120.1
C3—C2—C21	125.2 (3)	C24—C23—C22	121.6 (5)
C1—C2—C21	127.9 (3)	C24—C23—H23	119.2
C3—C2—Fe1	70.0 (2)	C22—C23—H23	119.2
C1—C2—Fe1	68.47 (19)	C23—C24—C25	119.0 (4)
C21—C2—Fe1	129.6 (3)	C23—C24—H24	120.5
C4—C3—C2	108.4 (4)	C25—C24—H24	120.5
C4—C3—Fe1	69.3 (2)	C26—C25—C24	120.3 (4)
C2—C3—Fe1	69.0 (2)	C26—C25—H25	119.9
C4—C3—H3	125.8	C24—C25—H25	119.9
C2—C3—H3	125.8	C21—C26—C25	121.1 (4)
Fe1—C3—H3	127.5	C21—C26—H26	119.5
C5—C4—C3	108.6 (3)	C25—C26—H26	119.5
C5—C4—Fe1	69.4 (2)	Cl2B—C30A—Cl1A	110.2 (5)
C3—C4—Fe1	69.8 (2)	Cl2B—C30A—H30C	109.6
C5—C4—H4	125.7	Cl1A—C30A—H30C	109.6
C3—C4—H4	125.7	Cl2B—C30A—H30A	109.6
Fe1—C4—H4	126.7	Cl1A—C30A—H30A	109.6
C4—C5—C1	108.1 (4)	H30C—C30A—H30A	108.1
C4—C5—Fe1	70.2 (2)	Cl2C—C30B—Cl1B	114.9 (7)
C1—C5—Fe1	68.9 (2)	Cl2C—C30B—H30D	113.2
C4—C5—H5	126.0	Cl1B—C30B—H30D	109.6
C1—C5—H5	126.0	Cl2C—C30B—H30B	108.5
Fe1—C5—H5	126.5	Cl1B—C30B—H30B	108.5
C10—C6—C7	108.7 (5)	H30D—C30B—H30B	101.0
C10—C6—Fe1	70.2 (3)		
			
N1—C11—C1—C2	92.4 (4)	N1—C11—C1—C5	−91.2 (4)

### Hydrogen-bond geometry (Å, º)

**Table:**

*D*—H···*A*	*D*—H	H···*A*	*D*···*A*	*D*—H···*A*
C11—H11*A*···I1^i^	0.99	3.17	3.988 (4)	140
C12—H12*C*···I1	0.98	3.21	4.117 (5)	154
C12—H12*B*···Cl2*C*	0.98	3.51	3.787 (7)	99
C13—H13*A*···I1	0.98	3.27	4.158 (5)	151
C14—H14*A*···I1	0.98	3.14	4.057 (5)	157
C14—H14*B*···I1^i^	0.98	3.25	4.077 (5)	143
C30*A*—H30*A*···I1^ii^	1.00	2.89	3.867 (7)	166
C13—H13*B*···CT2^iii^	0.98	2.98	3.901 (6)	158
C23—H23···CT1^iv^	0.95	2.69	3.600 (7)	160

Symmetry codes: (i) −*x*+1, *y*+1/2, −*z*+2; (ii) −*x*, *y*+1/2, −*z*+1; (iii) −*x*+2, *y*−1/2, −*z*+2; (iv) −*x*+1, *y*+1/2, −*z*+1.
